# Quantitative Analysis of the Influence of the Xiaolangdi Reservoir on Water and Sediment in the Middle and Lower Reaches of the Yellow River

**DOI:** 10.3390/ijerph20054351

**Published:** 2023-02-28

**Authors:** Xianqi Zhang, Wenbao Qiao, Yaohui Lu, Jiafeng Huang, Yimeng Xiao

**Affiliations:** 1 Water Conservancy College, North China University of Water Resources and Electric Power, Zhengzhou 450046, China; 2 Collaborative Innovation Center of Water Resources Efficient Utilization and Protection Engineering, Zhengzhou 450046, China; 3 Technology Research Center of Water Conservancy and Marine Traffic Engineering, Zhengzhou 450046, China

**Keywords:** xiaolangdi reservoir, the middle and lower reaches of the Yellow River, runoff and sediment transport, Mann-Kendall test, the wavelet analysis

## Abstract

The Xiaolangdi Reservoir is the second largest water conservancy project in China and the last comprehensive water conservancy hub on the mainstream of the Yellow River, playing a vital role in the middle and lower reaches of the Yellow River. To study the effects of the construction of the Xiaolangdi Reservoir (1997–2001) on the runoff and sediment transport in the middle and lower reaches of the Yellow River, runoff and sediment transport data from 1963 to 2021 were based on the hydrological stations of Huayuankou, Gaocun, and Lijin. The unevenness coefficient, cumulative distance level method, Mann-Kendall test method, and wavelet transform method were used to analyze the runoff and sediment transport in the middle and lower reaches of the Yellow River at different time scales. The results of the study reveal that the completion of the Xiaolangdi Reservoir in the interannual range has little impact on the runoff in the middle and lower reaches of the Yellow River and a significant impact on sediment transport. The interannual runoff volumes of Huayuankou station, Gaocun station, and Lijin station were reduced by 20.1%, 20.39%, and 32.87%, respectively. In addition, the sediment transport volumes decreased by 90.03%, 85.34%, and 83.88%, respectively. It has a great influence on the monthly distribution of annual runoff. The annual runoff distribution is more uniform, increasing the runoff in the dry season, reducing the runoff in the wet season, and bringing forward the peak flow. The runoff and Sediment transport have obvious periodicity. After the operation of the Xiaolangdi Reservoir, the main cycle of runoff increases and the second main cycle disappears. The main cycle of Sediment transport did not change obviously, but the closer it was to the estuary, the less obvious the cycle was. The research results can provide a reference for ecological protection and high-quality development in the middle and lower reaches of the Yellow River.

## 1. Introduction

The natural runoff processes of rivers are characterized by significant changes in abundance and periodicity [1]. These variations play a role in transporting materials, shaping habitats, and maintaining biological survival [2,3]. This is essential for maintaining river ecosystems [4,5,6]. However, with the development of society, people have built many hydraulic projects on rivers to meet their development needs. According to the International Commission on Dams, as of April 2020, the number of existing dams worldwide was 58,713, of which 23,841, or 40.6%, were in China [7]. These hydraulic projects have changed the natural hydrological form of the river while meeting the needs of the people [8,9,10,11]. Different hydraulic projects bring about different changes in the hydrological situation of the river [12]. In order to study the influence of hydraulic engineering on river hydrology, many scholars in China and abroad have conducted studies on this topic since the 20th century. Williams et al. conducted a study and analysis of 21 reservoirs in the U.S. The study found that flood flows downstream of the reservoirs were significantly lower, with the average flood flow after reservoir construction being only 45% of that before construction, and the average annual flow was also reduced by approximately 10% [13]. Walling and Fang concluded that reservoir use is an important factor influencing the amount of sediment entering the sea in recent years by analyzing the changes in the amount of sediment entering the world’s rivers in recent years and that the effect of climate change is limited [14]. Further analysis by Vorosmarty et al. showed that 40% of the world’s annual runoff and 25% of the volume of sediment entering the sea is impounded by reservoirs [15]. Song et al. analyzed the effect of reservoir storage on river conditions at the Chinese scale and found an overall moderate degree of change [16]. Li and Kong et al. analyzed the effects of different reservoir operations on the hydrological situation of the lower Yellow River based on the range-of-variation method and found that there were large differences in the ecohydrological characteristics under different operations [17,18]. The operation of the reservoirs has prevented river disconnection to some extent and changed the intra-annual distribution of runoff in and out of the reservoir stations, with a significantly lower percentage of water in the flood season [19]. Kong and Hu et al. analyzed the impact of reservoir water and sediment changes on the lower reaches of the Yellow River [20,21,22,23]. With the development of computers, the use of neural networks to analyze hydrological processes on the basis of large amounts of hydrological data has gained importance. Li Mengchu and Zhang Yanyan et al. used wavelet analysis to analyze the water sediment and flatland flow in the lower Yellow River on multiple time scales [24,25]. Most of these studies analyzed runoff and sediment transport variability on a large time scale and did not consider the analysis of river runoff and sediment transport variability on a monthly scale. Moreover, the methods used are mostly traditional hydrostatistical methods.

The Yellow River has the largest amount of mud and sediment in the world. It is characterized by less water and more sediment, and different sources of water and sediment [26,27]. The Xiaolangdi Reservoir, as the last controlling water hub in the seaward section of the Yellow River, has played a crucial role since its completion [28]. The hydrological impact of the Xiaolangdi Reservoir on the middle and lower reaches of the Yellow River is of great research value. Previous studies have been limited to the interannual variability of runoff and sediment transport. Moreover, the methods used are more traditional, and few of them have been combined with computers to analyze the hydrological patterns. In this paper, three important hydrological stations in the middle and lower reaches of the Yellow River are studied. Based on the flow data from 1963–2021, the influence of the Xiaolangdi reservoir on the runoff and sediment transport in the middle and lower reaches of the Yellow River was analyzed by combining traditional hydrological methods with computer applications, using the cumulative flat distance method, MK test method, and wavelet analysis. To study the hydrological changes in the middle and lower reaches of the Yellow River after the operation of the Xiaolangdi Reservoir. By comparing the changes in runoff and sediment transport in the middle and lower reaches of the Yellow River before and after the operation of the Xiaolangdi reservoir, the impact of the Xiaolangdi reservoir on the hydrological conditions of the downstream river is discussed. It provides guidance for the operation and scheduling of the Xiaolangdi Reservoir and also provides a reference for the construction and development of other reservoirs.

## 2. Study Area and Data Source

**Figure 1:**
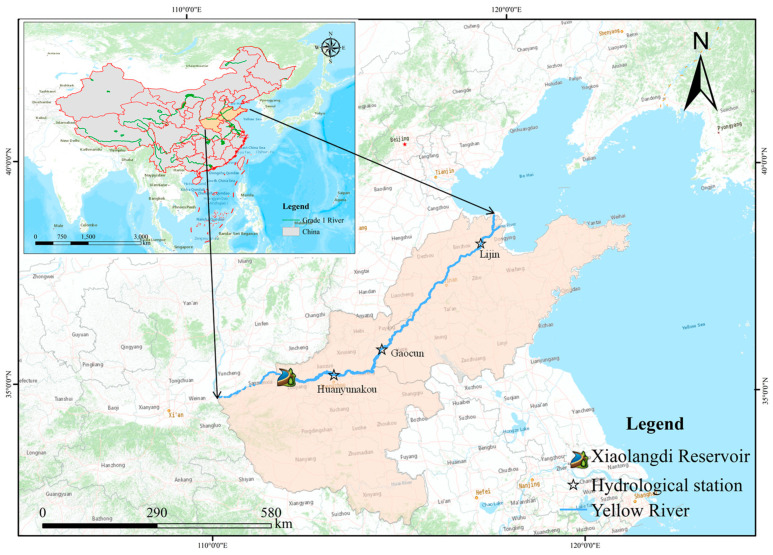
Location map of the study area.

The Xiaolangdi Reservoir is located between Luoyang City and Jiyuan City in Henan Province, controlling a watershed area of 694,000 km^2^. It is the last large comprehensive water conservancy project on the mainstream of the Yellow River and is a key project in the management and development of the Yellow River, which was completed in 2001 [29,30]. The middle and lower reaches of the Yellow River are mainly located in Henan and Shandong provinces, with a total length of 277 km. To study the influence of the construction of the Xiaolangdi reservoir on the variation of water and sediment in the lower reaches of the Yellow River, the runoff data and sediment concentration data of Huayuankou, Gaocun, and Lijin hydrological stations were selected in this study, and their positions are shown in Figure 1. The base map in Figure 1 is derived from ArcGis online map [31].

Data were obtained from the Yellow River Water Resources Bulletin, the Yellow River Sediment Bulletin, and the China River Sediment Bulletin.

## 3. Research Methods

### 3.1. Unevenness Coefficient

The unevenness coefficient is essentially the variation coefficient commonly used in hydrological statistics, which describes the dispersion degree between the average discharge of each period and the average discharge of the total period, that is, it reflects the unevenness degree of runoff distribution within a period [32].

(1) $$C_{v}=\frac{\delta }{\bar{R}}$$

(2) $$\bar{R}=\frac{1}{n}\sum_{i=1}^{n}R_{i}$$

(3) $$\delta =\sqrt{\frac{1}{n}\sum_{i=1}^{n}{\left(R_{i}-\bar{R}\right)}^{2}}$$

where $C_{v}$ is the unevenness coefficient, $R_{i}$ is the average runoff in each period, $\bar{R}$ is the average runoff in the total period, *δ* is the mean square error of the runoff series in the total period, and n is the number of traffic sequences.

### 3.2. Cumulative Anomaly

The cumulative anomaly method can visually reflect the anomaly results on the chart through the curve, to judge the changing trend of annual runoff and sediment transport [33,34]. For the hydrological element series, the cumulative anomaly at a certain time t is expressed as follows.

(4) $$x=\sum_{i=1}^{n}\left(x_{i}-\bar{x}\right)$$

(5) $$\bar{x}=\frac{1}{n}\sum_{i=1}^{n}x_{i}$$

where $x_{i}$ is the actual data at a certain time, $\bar{x}$ is the average value of a time series, and x is the cumulative flat distance value.

### 3.3. Two-Sample F-Test

Two-sample F-test was used to compare the variance of two normal populations [35]. Set $X\sim N\left(a_{1},\sigma_{1}^{2}\right)$ and $Y\sim N\left(a_{2},\sigma_{2}^{2}\right)$, and build $H_{0}\sim \sigma_{1}^{2}=\sigma_{2}^{2}$. The statistics are shown below.

(6) $$F=\frac{S_{1}^{2}}{S_{2}^{2}}$$

(7) $$S_{1}^{2}=\frac{1}{n_{1}-1}\sum_{i=1}^{n_{1}}{\left(X_{i}-\bar{X}\right)}^{2}$$

(8) $$S_{2}^{2}=\frac{1}{n_{2}-1}\sum_{i=1}^{n_{2}}{\left(Y_{i}-\bar{Y}\right)}^{2}$$

Generally, under the condition that $H_{0}$ holds, *F* follows the *F* distribution with degrees of freedom $n_{1}-1,n_{2}-1$. From the given α=0.05, F1−α2 and Fα2 satisfying the following relation can be obtained through the F distribution table.

(9) P=F<F1−α2=α2

(10) P=F>F1−α2=α2

If the calculated *F* value satisfies F<F1−α2 or  F>F1−α2, the null hypothesis $H_{0}\;$ is rejected, which indicates that the variance of the two groups of samples is significantly different. Otherwise, $H_{0}$ is accepted, indicating that there is no significant difference in the variance between the two groups of samples [36].

### 3.4. Mann-Kendall Test

The Mann-Kendall nonparametric test is a nonparametric statistical test method, which is often used in the mutation test and analysis of time series [37]. For a time-series *x* with *n* sample sizes, construct an order column $d_{k}=\overset{k}{\underset{i=1}{\sum }}r_{i}\left(2\leq k\leq n\right)$, where $r_{i}$ denotes the cumulative value of the *i* sample $x_{i}$ greater than $x_{j}$ (1 ≤ *j* ≤ *i*).

(11) $$E\left[d_{k}\right]=\frac{k\left(k-1\right)}{4}$$

(12) $$V_{ar}\left[d_{k}\right]=\frac{k\left(k-1\right)\left(2k+5\right)}{72},2\leq k\leq n$$

Under the assumption of random independence of the time series, the statistic is defined as follows.

(13) $$UF_{k}=\frac{d_{k-}E\left[d_{k}\right]}{\sqrt{V_{ar}\left[d_{k}\right]}}\;k=1,2,3\cdots n$$

At a given level of significance ($x=0.05,U_{0.05}=\pm 1.96$) conditions, when $UF_{k}>U_{\alpha }$, show that the sequence has an obvious trend of increase or decrease. If $UF_{k}$ is greater than 0, it indicates that the series shows an upward trend; otherwise, it indicates a downward trend. When the curve of the statistic exceeds the critical line, it indicates a significant upward or downward trend. By applying the same method to the inverse sequence, the curve $UB_{k}$ can be obtained. If the $UF_{k}$ and $UB_{k}$ curves intersect, and the intersection point is between the critical line, the time corresponding to the intersection point becomes the time at which the mutation begins [38].

### 3.5. Continuous Wavelet Analysis

Hydrological system change is a complex process, and the multi-time scale is an important feature in the process of hydrological time series change [39]. Wavelet analysis is used to analyze the time scale of the time series data in the study area, to accurately find out the period of the time series data and judge the stage of each period. In this paper, Morlet is selected to analyze long sequences [40].

Wavelet transform: Wavelet function refers to a class of functions with oscillating characteristics and can quickly decline to zero [41]. The expression is as follows.

(14) $$\int_{-\infty }^{+\infty }\psi \left(t\right)dt=0$$

Let $R{\;}^{2}$ denote the space of square-integrable functions measurable on the real number line. For signal $f\left(t\right)$ belonging to $R{\;}^{2}$, its continuous wavelet transform (CWT) is defined as follows.

(15) $$W_{f}\left(a,b\right)=\langle f,\psi_{a,b}\rangle =\frac{1}{\sqrt{\left|a\right|}}\int_{R}^{\;}f\left(t\right)\psi (\frac{t-b}{a})dt$$

where, $W_{f}\left(a,b\right)$ is called the coefficient of wavelet transform; *a* is the scale factor; *b* stands for the time factor. The wavelet coefficient can simultaneously reflect the characteristics of time and frequency domain parameters *a* and *b*, which is the output of time series $f\left(t\right)$ through unit impulse response [42,43].

Wavelet square difference: the wavelet square difference can be obtained by integrating the square of all the wavelet transform coefficients in the time domain concerning the time scale *a*. For discrete time series, its relation is as follows.

(16) $$V\left(a\right)=\frac{1}{n}\sum_{b=1}^{n}w_{f}^{2}\left(a,b\right)$$

where, $W_{f}\left(a,b\right)$ is the coefficient of $f\left(t\right)$ at position *a* and *b*, *n* is the number of samples of the sequence, the extreme value of wavelet square difference corresponds to the significant period, and when the wavelet coefficient reaches the maximum value, the corresponding wavelet scale matches the sequence period best [44,45].

## 4. Analysis and Discussion

### 4.1. Interannual Runoff Analysis

**Figure 2:**
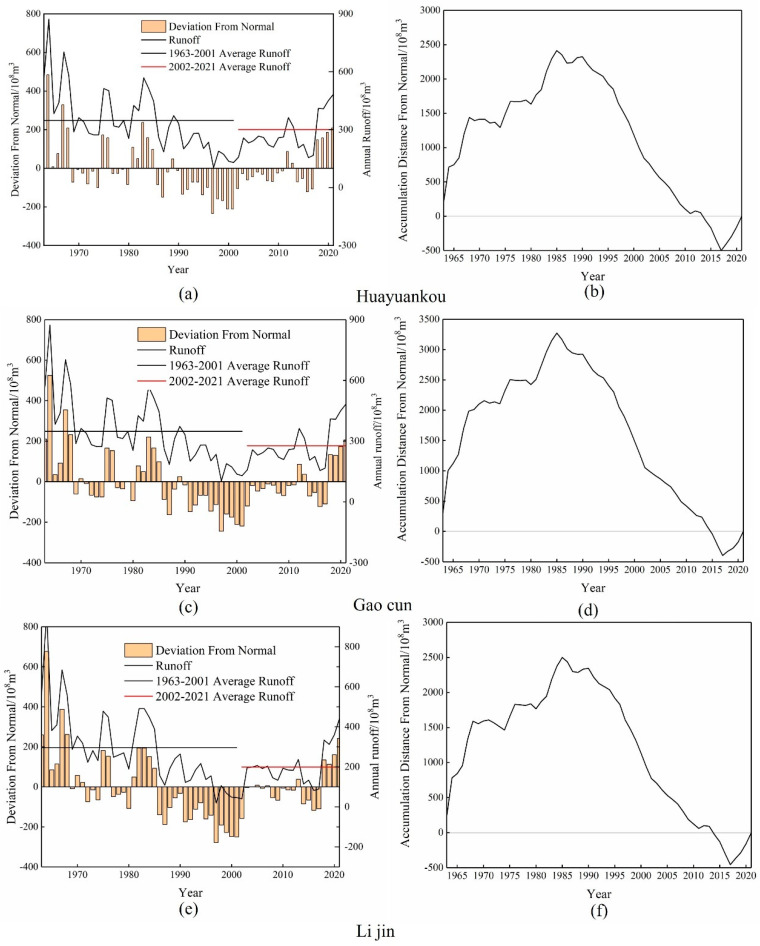
Changes in runoff volume and cumulative flat distance from 1963-021. (**a**,**c**,**e**) represent the runoff change of the three hydrological stations, and (**b**,**d**,**f**) represent the cumulative level distance of the three hydrological stations.

The three main hydrological stations of Huayuankou, Gaocun, and Lijin in the middle and lower reaches of the Yellow River from 1963 to 2021, the measured annual runoff change curves and their cumulative distances are shown in Figure 2 and Table 1. To study the impact of the Xiaolangdi reservoir on the middle and lower reaches of the Yellow River, the study sequence was divided into 1963–2001 and 2002–2021.

According to Figure 2 and Table 1, the interannual variation trend of runoff at the three hydrological stations in the lower Yellow River is relatively close, and the interannual variation of runoff is synchronized. From 1963 to 2021, the annual runoff in the middle and lower reaches of the Yellow River showed a large fluctuation, with the overall trend of decreasing first and then increasing, decreasing from 1963 to 2017 and increasing from 2017 to 2021. The annual minimum occurred in 1997, with a runoff of 14.26 $\times 10^{9}$ m^3^, 10.34 $\times 10^{9}$ m^3^ and 1.861 $\times 10^{9}$ m^3^, respectively. The annual maximum runoff occurred in 1964, which was 86.11 $\times 10^{9}$ m^3^, 87.29 $\times 10^{9}$ m^3^ and 97.31 $\times 10^{9}$ m^3^, respectively. The average annual runoff of Huayuankou, Gaocun, and Lijin hydrological stations is respectively 35.088 $\times 10^{9}$ m^3^, 32.446 $\times 10^{9}$ m^3^ and 26.268 $\times 10^{9}$ m^3^. The unevenness coefficient was 0. 40, 0.45, and 0.66, respectively. As a whole, the unevenness coefficient of the three main hydrological stations in the lower reaches of the Yellow River is relatively large. Considering the geographical location of the three hydrological stations, it can be seen that the closer the station is to the estuary, the smaller the annual runoff, and the biggest fluctuation of the annual runoff.

It can be seen from Table 1 that the middle and lower reaches of the Yellow River are divided into wet years and dry years, and the difference between the maximum and minimum annual runoff is about three times. The interannual variation in abundance and drought characteristics of Huayuankou, Gaocun, and Lijin hydrological stations are the same. In the 1960s, 1970s, and 1980s, it was in partial abundance. From the 1990s to the early 21st century, it was in a state of decline. According to Figure 2b,d,f, the annual runoff of the middle and lower reaches of the Yellow River from 1963 to 2021 can be divided into two significant water-rich sections, namely, 1963 to 1989 and 2017 to 2021. There were two significant flat water sections, namely 1968~1973 and 1976~1979. A significant dry period is from 1989 to 2017. In the 1990s, the decrease of the Yellow River runoff was aggravated, and the most serious runoff interruption occurred in the history of the Yellow River during this period: 122 days in 1995, 136 days in 1996, and 226 days in 1997 [46]. The situation eased after the Xiaolangdi Reservoir was put into operation.

**Figure 3:**
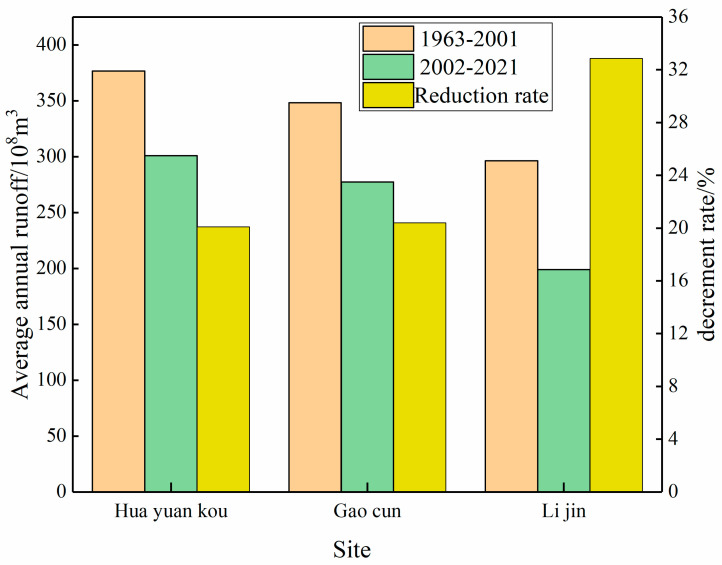
Average annual runoff and runoff decrement rate before and after the construction of the Xiaolangdi Reservoir.

Figure 3 shows that from 1963 to 2001 (before the operation of the reservoir), the average annual runoff of Huayuankou, Gaocun, and Lijin hydrological stations were 37.653 $\times 10^{9}$ m^3^, 34.837 $\times 10^{9}$ m^3^ and 29.636 $\times 10^{9}$ m^3^, respectively. From 2002 to 2021 (after the operation of the reservoir), the average annual runoff of Huayuankou, Gaocun, and Lijin hydrological stations were 30.086 $\times 10^{9}$ m^3^, 27.731 $\times 10^{9}$ m^3^ and 19.896 $\times 10^{9}$ m^3^, respectively. The average annual runoff of the three hydrological stations was reduced by 20.1%, 20.39%, and 32.87%, respectively, at Huayuankou, Gaecun, and Lijin hydrological stations. The reduction rates of Huayuankou station and Gaocun station are the same, and the reduction rate of the Lijin hydrology station is the largest. After the operation of the Xiaolangdi reservoir, the unevenness coefficient of the annual runoff of the three hydrological stations decreased compared with that before the operation, indicating that the fluctuation of annual runoff decreased after the operation of the Xiaolangdi reservoir.

To quantitatively assess the variation trend of runoff in the lower Yellow River, the Mann-Kendall trend test was used for quantitative analysis. The Mann–Kendall nonparametric test results of annual runoff in statistical series of hydrological stations along the lower Yellow River are shown in Table 2.

According to Table 2, from the time series analysis from 1963 to 2021, the Zc values of Huayuankou, Gaocun, and Lijin stations are all negative, and the absolute value is greater than 2.32, which passes the significance test at 99% level. From the perspective of interannual variation, the runoff is in a downward trend, the decline degree of Gaocun station is small, and the decline degree of Lijin station is the largest. From the time series analysis of 1963–2001 before the construction of the Xiaolangdi Reservoir, the absolute values of the three stations were also greater than 2.32, and the runoff was in a downward trend, especially in Lijin station, which was consistent with the changing trend from 1963 to 2021. From the analysis of the time series from 2002 to 2021 after the construction of the Xiaolangdi, the Zc values of the three stations are all positive and less than 2.32. From the perspective of interannual, the runoff of the three hydrological stations is on the rise after the completion of the Xiaolangdi reservoir.

It is considered that the interannual discharge series of the three hydrological stations have different trends before and after the Xiaolangdi construction. Based on a two-sample F-test analysis, the difference in annual average runoff of Huayuankou station, Gaocun station, and Lijin Station before and after the construction and operation of the reservoir was tested, and the statistical results are listed in Table 3. According to Table 3, there is no significant difference in the annual runoff between the three hydrological stations before and after the Xiaolangdi reservoir operation. This indicates that during the operation period of the Xiaolangdi Reservoir (2002–2021), although the annual runoff variation trend of Huayuankou station, Gaocun station, and Lijin station is opposite, it is not significant. It also indicates that the Xiaolangdi Reservoir has limited influence on the interannual runoff variation process of the middle and lower reaches of the Yellow River.

### 4.2. Intra-Annual Runoff Characterization

**Figure 4:**
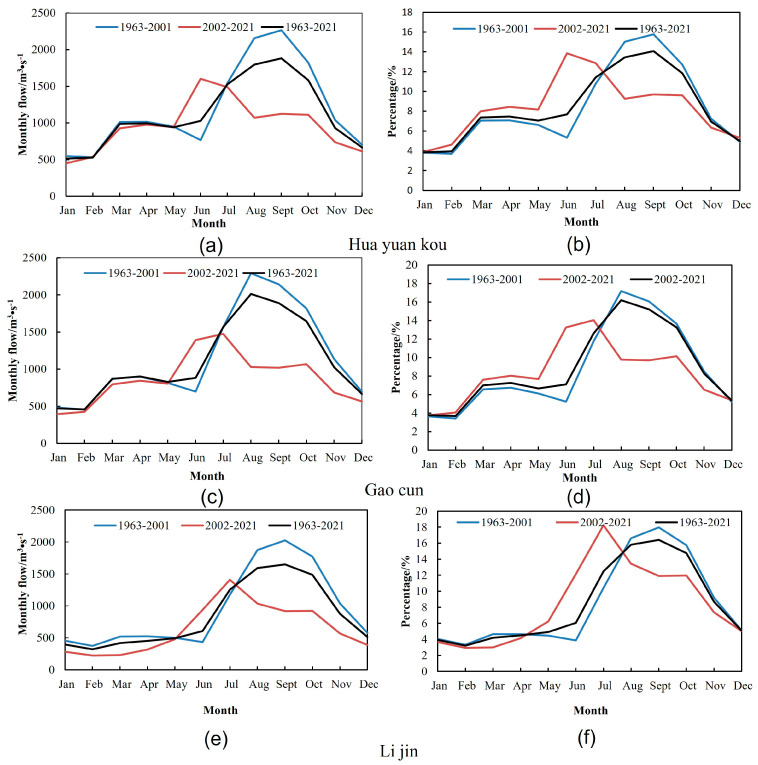
The annual proportion of monthly average discharge and runoff before and after the construction of the Xiaolangdi reservoir. (**a**,**c**,**e**) are the annual flow variation of the three hydrological stations, and (**b**,**d**,**f**) are the annual flow proportion.

According to three different periods from 1963 to 2001, 2002 to 2021, and 1963 to 2021, the monthly flow process lines of Huayuankou station, Gaocun station, and Lijin station for many years and the annual proportion of each month are drawn respectively, as shown in Figure 4. It can be known from the annual discharge process line that the annual discharge distribution in the middle and lower reaches of the Yellow River is quite different, and there are obvious wet and dry periods. Before the operation of the Xiaolangdi reservoir, the monthly average discharge of Huayuankou station, Gaocun station, and Lijin station in September was the largest, which were 2264.17 m^3^/s, 2114.47 m^3^/s, and 2026.73 m^3^/s, respectively, accounting for 15.78%, 15.85% and 17.96% of the whole year. After the operation of the reservoir, the maximum months of the three hydrological stations are June, July, and July, and the maximum flows are 1602.64 m^3^/s, 1474.32 m^3^/s, and 1408.33 m^3^/s, accounting for 13.85%, 14.03%, and 18.22% of the whole year, respectively. Before the operation of the reservoir, the wet season of Huayuankou station, Gaocun station, and Lijin Station accounted for 54.29%, 54.93%, and 60.72% of the whole year, respectively; after the operation of the Xiaolangdi reservoir, the wet season of the three hydrological stations accounted for 41.41%, 43.66%, and 55.49% of the whole year, respectively. The reservoir has changed the proportion of runoff in the wet season to some extent, but the flow in the wet season is still large. After the construction of the reservoir, the maximum monthly discharge was greatly reduced, and the minimum discharge was slightly reduced but not significantly.

**Figure 5:**
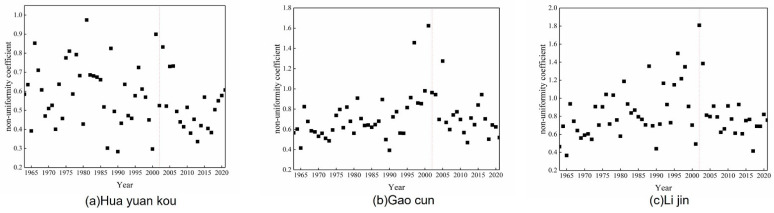
Unevenness coefficient of annual runoff from 1963 to 2021. (**a**,**b**,**c**) are Huayuankou Station, Gaocun Station and Lijin Station respectively.

To analyze the influence of the operation period of the Xiaolangdi reservoir construction on the annual runoff distribution, two indicators, the annual runoff unevenness coefficient of the long series from 1963 to 2021 of the three hydrological stations and the monthly distribution ratio difference before and after the construction and operation of the reservoir, was selected to analyze the variation rules. The analysis results are shown in Table 4 and Figure 5. According to the statistical results in Table 4, Huayuankou station and Gaocun station increased their annual proportion from January to April, while Lijin Station did the opposite, but the change was not significant. The proportion of the three stations increased in May and June, and decreased in August, September, and October, with the biggest change in June and August. This is because the flood season is approaching, and the Xiaolangdi Reservoir will increase its discharge in May and June to prepare for flood prevention. According to Figure 5, after the construction of the Xiaolangdi is completed, the fluctuation range of the unevenness coefficient during 2002−2021 is small, indicating that the annual distribution of runoff is more uniform. After the operation of the Xiaolangdi reservoir, it has had a great influence on the annual runoff distribution, which is mainly manifested as increasing the discharge in the dry season and decreasing the discharge in the wet season. It plays the role of flood control and storage in the lower reaches of the Yellow River, alleviates the flood pressure in the middle and lower reaches of the Yellow River in flood season, and also maintains the flow demand to maintain the normal ecological operation of the river and promotes the ecological restoration of the lower reaches [47].

### 4.3. Characterization of Sediment Transport Volume

**Figure 6:**
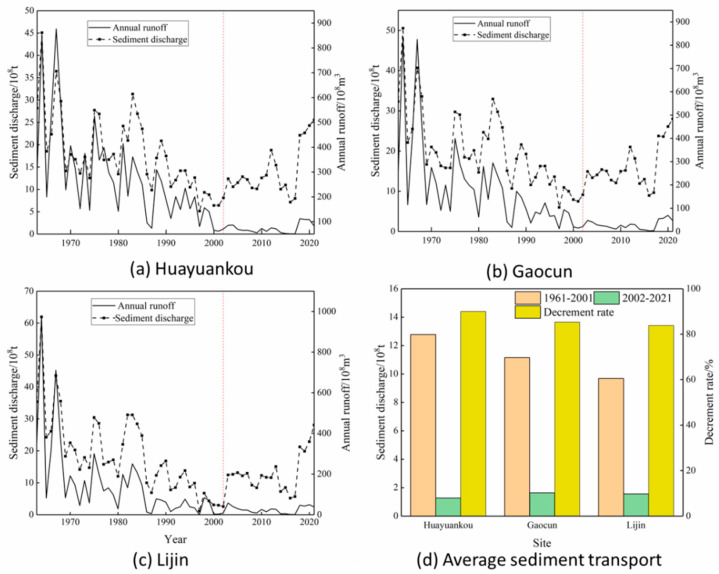
Variation in runoff and sediment transport and the multi-year average of sediment transport. (**a**–**c**) are the changes of runoff and sediment volume of the three hydrological stations respectively, and (**d**) are the annual average values of the three hydrological stations.

To describe the relationship between annual runoff and sediment transport in the middle and lower reaches of the Yellow River, the relationship curves between annual runoff and sediment transport at three major hydrological stations in the middle and lower reaches of the Yellow River are depicted in Figure 6 below.

As can be known from Figure 6, both annual runoff and sediment transport show a decreasing trend, and before the operation of the Xiaolangdi Reservoir, sediment transport, and runoff changed in the same trend and fluctuate widely. After the operation of the Xiaolangdi Reservoir, the water-sediment relationship in the middle and lower reaches of the Yellow River is out of balance, and there is volatility in the annual runoff, while the value of sediment transport is relatively stable. The average sediment transport in the middle and lower reaches of the Yellow River has been greatly reduced. The amount of sediment transported at Huayuankou station decreased from 1278 $\times 10^{6}$ t to 127 $\times 10^{6}$ t, a reduction of 90.03%, at Gaocun station from 1116 $\times 10^{6}$ t to 164 $\times 10^{6}$ t, a reduction of 85.34%, and at Lijin Station from 968 $\times 10^{6}$ t to 156 $\times 10^{6}$ t, a reduction of 83.88%. This is because the primary role of the Xiaolangdi reservoir is to reduce silting, especially in the early stage of reservoir operation, 7.5 $\times 10^{9}$ m^3^. Silting and sediment storage capacity can be used to greatly reduce the incoming sediment into the lower Yellow River [48].

### 4.4. Analysis of Sudden Changes in Runoff and Sediment Transport

**Figure 7:**
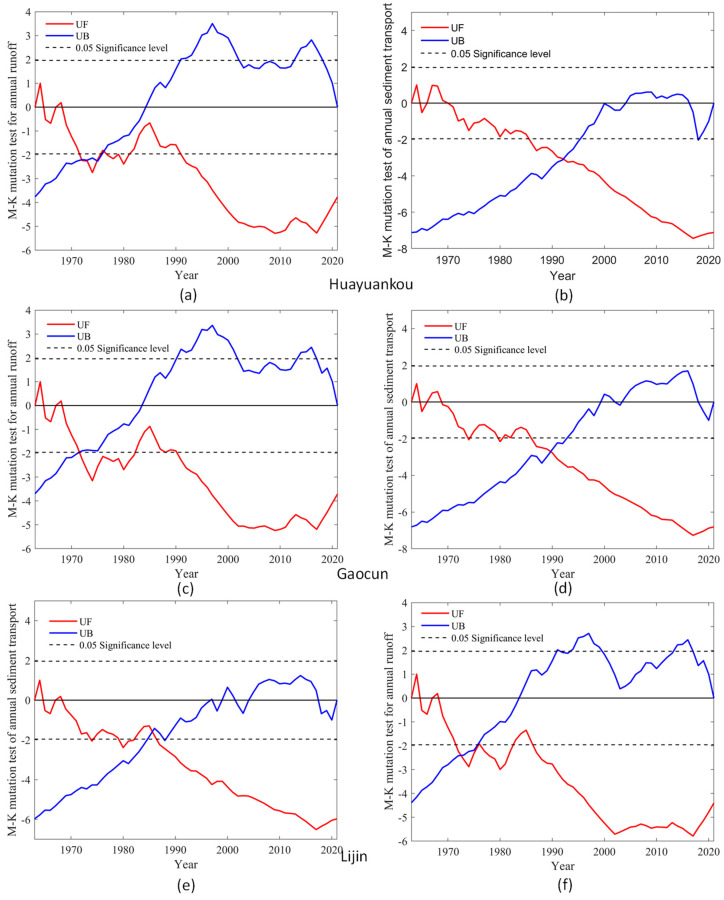
Mutation tests of annual runoff and Sediment transport (**a**,**c**,**e**) are mutation tests of runoff, (**b**,**d**,**f**) are mutation tests of Sediment transport.

The Mann–Kendall mutation test analysis was performed for runoff and sediment transport at three hydrological stations in the middle and lower reaches of the Yellow River. The results of the analysis are shown in Figure 7.

It can be seen from Figure 7a that the annual runoff of Huayuankou station, Gaocun station, and Lijin Station showed a decreasing trend in all periods except 1963 and 1964. At 0.05 confidence level, Huayuankou station in 1972–1981 and 1990–2021 exceeded the threshold value of significance level 0.05, and the runoff reduction trend intensified. The annual runoff changed dramatically in 1972 and 1976. As seen in Figure 7c, the abrupt change test is broken at the Takamura station from 1973–1983 and 1990–2021, indicating a significant trend of decreasing runoff during that period, and the existence of an abrupt change point in 1973, indicating an abrupt change in runoff in 1973. As shown in Figure 7e, the Lijin station passed the mutation test in 1980, 1988–2021, and there is a mutation point in 1987, indicating that the runoff from the Lijin station was mutated in 1987.

As shown in Figure 7b, the overall sediment transport at the Garden Mouth and Takamura stations is high in 1963–1964 and 1967–1969, and the UF is negative from 1969 to 2021, with a decrease in annual sediment transport. The trend in sediment transport at the Lijin station increases from 1963–1964, and the UF is negative from 1967–2021, indicating a decreasing trend in sediment transport during that period. The trend of decreasing sediment transport at the Garden Mouth Station intensified after 1985, with a sudden change in 1992. As can be seen from Figure 7d, Takamura station passed the significance test at the 0. 01 level in 1985, with a significant trend of decreasing sediment transport. There is a clear intersection in 1989 indicating an abrupt change in sediment transport from Takamura in 1987. As can be seen from Figure 7f, the Lijin station passed the abrupt change test in 1974–1983 and 1985–2021, indicating an increasing trend of decrease during that period. The presence of two mutation points in 1974 and 1976 indicates that sediment transport at the Lijin station underwent abrupt changes in 1974 and 1976.

Considering the Mann-Kendall abrupt change test of annual runoff and sediment transport at three hydrological stations in the middle and lower reaches of the Yellow River, it can be seen that the runoff and sediment transport were increasing trends in the early 1960s and decreasing trends after the 1960s. The UF trend for runoff is generally consistent, indicating that tributaries in the middle and lower reaches have limited influence and that the largest influence on runoff remains upstream of incoming water. The trend of UF values of sediment transport at Huayuankou station and Gaocun stations are consistent, and the trend of UF values at Lijin station is close to the level in 2001–2017, and Huayuankou station and Gaocun station are still decreasing trend in that period. It indicates that the sediment transport in this section of the river from Takamura station to Lijin station has increased. The sudden change in runoff volume is not synchronized with the sudden change in sediment transport volume; the sudden change in sediment transport volume at Huankou and Gaocun stations lags behind the sudden change in runoff volume, and the sudden change in sediment transport volume at Lijin station takes precedence over the sudden change in runoff volume. After the completion of the Xiaolangdi Reservoir, there are no abrupt change points in runoff and sediment delivery, indicating that there are no major fluctuations in runoff and sediment volume. The UF curves for runoff slowed down at the rate of decrease, and the UF curves for sediment transport showed little change except for an obvious slowing trend in Lijin, and the UF curves for both runoff and sediment transport showed an increasing trend after 2017, indicating that the runoff and sediment transport to the sea in the middle and lower reaches of the Yellow River tended to increase.

### 4.5. Periodic Analysis of Runoff and Sediment Transport

**Figure 8:**
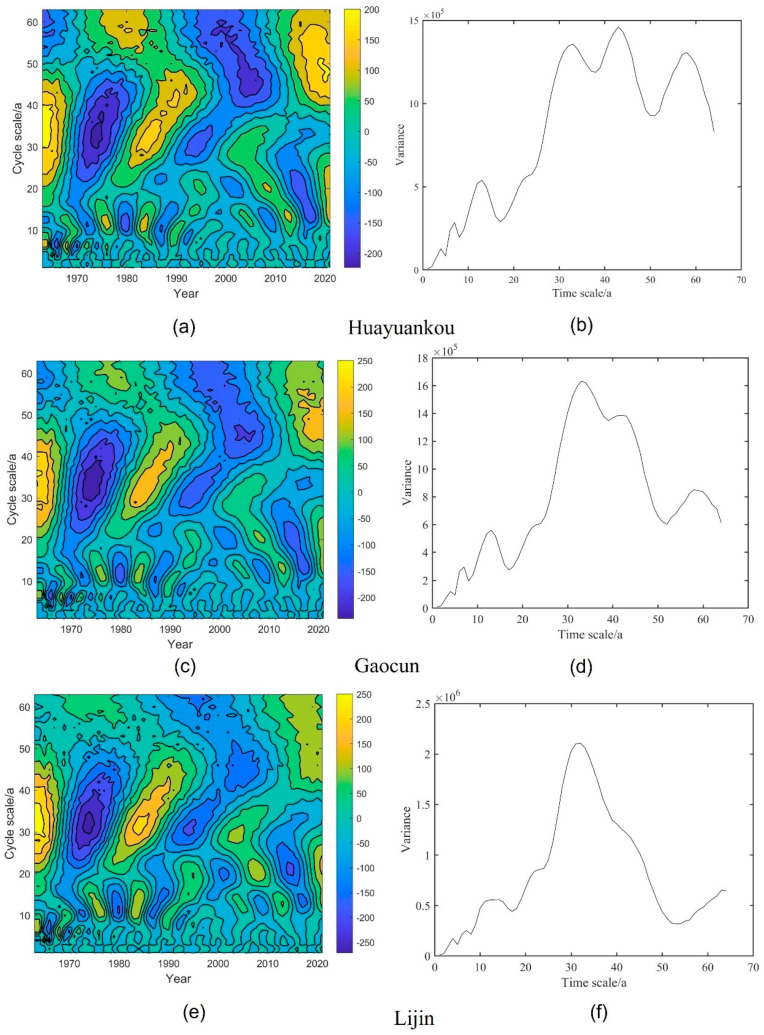
Contour and variance maps of runoff from three hydrological stations in the middle and lower reaches of the Yellow River. (**a**,**c**,**e**) are contour maps of three stations, while (**b**,**d**,**f**) are variance maps.

**Figure 9:**
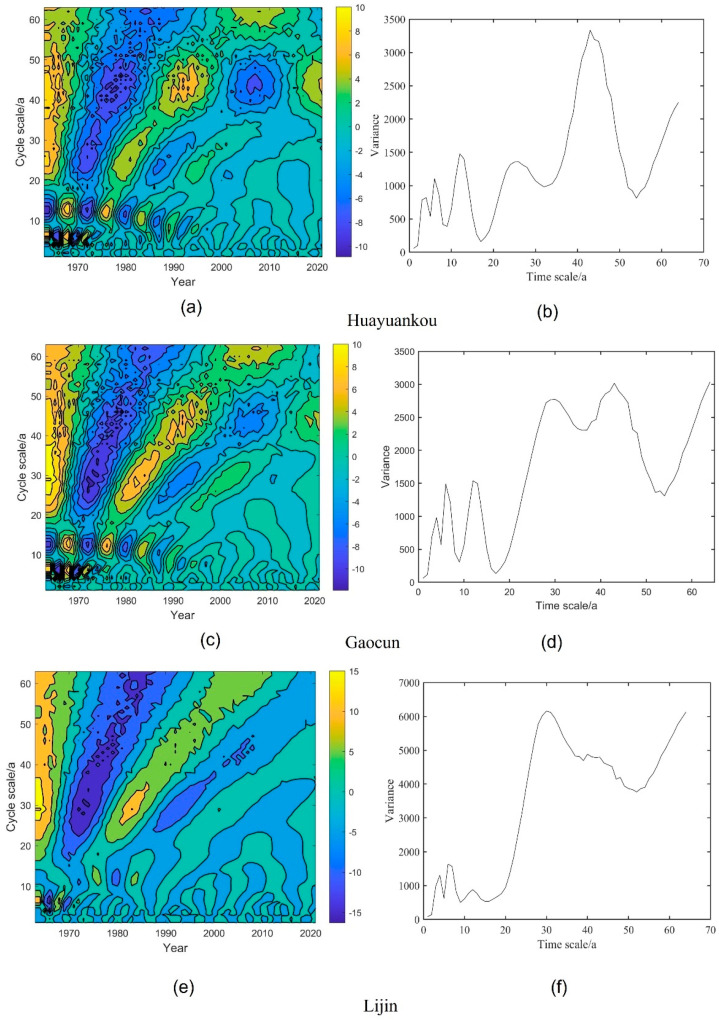
Contour and variance maps of sediment transport in the middle and lower reaches of the Yellow River. (**a**,**c**,**e**) are isoline maps of the three stations, while (**b**,**d**,**f**) are three variance maps.

Morlet wavelets were used to analyze the long series of runoff and sediment transport in the middle and lower reaches of the Yellow River periodically. The wavelet analysis contours and wavelet variance variations are shown in Figure 8 and Figure 9.

From Figure 8, it can be seen that the three hydrological stations in the middle and lower reaches of the Yellow River have different scales of cyclic variation, and there are relatively obvious variations in abundance and depletion years. The cycles of runoff at Garden Mouth and Takamura stations are mainly 58a, 43a, 32a, 12a, 8a, and 3a, with the 32a extreme point being the largest, indicating the most intense cycle oscillations and the most pronounced cycles on the 32a scale. The Lijin station cycle is mainly 32a, 12a, 8a, and 3a, with the main cycle being 32a. There is an alternating “abundance-depletion-abundance-depletion-abundance” on the main cycle scale. Wavelet analysis of runoff data from 1963–2021 at three major hydrological stations in the middle and lower reaches of the Yellow River shows that overall, the center of the main cycle low contour in the middle and lower reaches of the Yellow River is located in 1973 and 2000. The operation of the Xiaolangdi Reservoir changed the cycle of runoff, with the main cycle of 32a accompanied by sub-cycles of 12a, 8a, and 3a during the period 1963–2001. After the operation of the Xiaolangdi Reservoir, the main cycle is 58a and the cycle fluctuates in a wide range, and some of the sub-main cycles disappear. The contours of high annual runoff have not yet closed, indicating that annual runoff will be on the high side for some time to come.

As shown in Figure 9, there are multiple cycles of sediment transport in the middle and lower reaches of the Yellow River, mainly 43a, 28a, 15a, 8a, and 3a in Huayuankou station and Gaocun station, and 28a, 12a, 8a, and 2a in Lijin station. Sediment transport undergoes a cycle of “more–less–more–less–more” on the main cycle scale. Overall, the low contour of sediment transport in the middle and lower Yellow River is located between 1972 and 2005. After the construction of the Xiaolangdi, the main cycle of Garden Mouth and High Village remained at 43a, and the second main cycle disappeared. The closer to downstream, the less pronounced and more volatile the cycle. The high contour of the sediment transport bias is not closed, indicating that sediment transport will increase next.

## 5. Conclusions

In this paper, the effects of the operation of the Xiaolangdi Reservoir on the runoff and sediment transport rate of the middle and lower reaches of the Yellow River were analyzed from different scales using the inhomogeneous coefficients, the cumulative flat distance method, the Mann-Kendall test, and the wavelet analysis, and the following main conclusions were drawn.

(1) On the interannual scale, the operation of the Xiaolangdi Reservoir has a limited impact on interannual runoff and a significant impact on sediment transport. The annual runoff at Huayuankou, Gaocun, and Lijin stations decreased by 20.1%, 20.39%, and 32.87%, respectively, and the sediment transport decreased by 90.03%, 85.34%, and 83.88%, respectively. The closer to the mouth of the Yellow River, the greater the rate of reduction in runoff and the smaller the rate of reduction in sediment transport.
(2) On the intra-annual scale, the impact of the Xiaolangdi Reservoir on runoff is greater. Regulates the distribution of runoff during the year, mainly by cutting peak runoff, reducing runoff during the abundant period, and increasing runoff during the dry period. The ratio of runoff during the abundant water period to the whole year was reduced, and the ratio of abundant water period to the whole year was reduced from 54.29%, 54.93%, and 60.72% to 41.41%, 43.66%, and 55.49% at Huayuankou station, Takamura Station, and Lijin Station. The timing of the maximum flow occurring during the year was also changed, with the maximum flow occurrence being adjusted from September to June and July.
(3) The Mann-Kendall test shows that the tributaries of the middle and lower reaches of the Yellow River do not have a significant impact on interannual runoff. The overall runoff and sediment transport in the middle and lower Yellow River from 1963 to 2021 shows a decreasing trend with abrupt and unsynchronized changes. The operation of the Xiaolangdi Reservoir has mitigated this downward trend to some extent.
(4) By wavelet analysis, the runoff cycles in the middle and lower reaches of the Yellow River are runoff cycles 58a, 43a, 32a, 12a, 8a, and 3a, and the main cycles are 43a and 32a; the sediment transport cycles are runoff cycles of 43a, 28a, 15a, 8a, 3a, and the main cycles are 43a and 28a. The operation of the Xiaolangdi Reservoir has changed the main cycle of annual runoff to some extent, causing the disappearance of the second main cycle, but the effect on the main cycle of sediment transport is not significant, and the cycle is less pronounced the closer it is to the mouth of the sea. The contours of the larger values of the main cycle of runoff and sediment transport did not close, indicating that runoff and sediment transport will increase next.

## Figures and Tables

**Table 1:** Statistical characteristics of interannual runoff variation.

Site	Year	Average Value/10^8^ m^3^	Nonuniform Coefficient	Distance from Mean/%
Huayuankou station	1963–1969	550.03	0.32	322.55
	1970–1979	380.73	0.23	11.15
	1980–1989	412.77	0.28	96.24
	1990–1999	256.88	0.23	−317.77
	2000–2009	231.57	0.17	−384.98
	2010–2019	303.54	0.31	−193.85
	2020–2021	498.40	0.02	64.73
	1963–2001	376.53	0.41	7.31
	2002–2021	300.86	0.33	−14.26
	1963–2001	350.88	0.40	
Gaocun station	1963–1969	546.30	0.34	479.34
	1970–1979	352.57	0.24	87.31
	1980–1989	373.90	0.31	153.09
	1990–1999	222.99	0.28	−312.31
	2000–2009	211.15	0.23	−348.83
	2010–2019	276.69	0.32	−146.70
	2020–2021	467.10	0.03	88.10
	1963–2001	348.34	0.46	7.43
	2002–2021	277.31	0.34	−14.48
	1963–2001	324.26	0.45	
Lijin station	1963–1969	550.03	0.38	765.75
	1970–1979	311.22	0.27	184.79
	1980–1989	305.40	0.45	162.64
	1990–1999	140.76	0.48	−464.15
	2000–2009	141.14	0.47	−462.69
	2010–2019	186.20	0.44	−291.16
	2020–2021	400.35	0.10	104.82
	1963–2001	296.36	0.65	12.82
	2002–2021	198.96	0.49	−24.26
	1963–2001	262.68	0.66	

**Table 2:** Mann-Kendall trend analysis of annual runoff downstream of Yellow mouth.

Site	1963–2021			1963–2001			2002–2021		
	Zc	Judgment	Trend	Zc	Judgment	Trend	Zc	Judgment	Trend
Huayuankou station	−3.75	|Zc| > 2.32	-	−4.6	|Zc| > 2.32	-	1.98	|Zc| < 2.32	+
Gaocun station	−3.69	|Zc| > 2.33	-	−4.83	|Zc| > 2.33	-	1.85	|Zc| < 2.33	+
Lijin station	−4.39	|Zc| > 2.34	-	−5.46	|Zc| > 2.34	-	0.94	|Zc| < 2.34	+

Note: Zc is the calculated value of the Mann-Kendall trend analysis.

**Table 3:** Statistical results of variance test.

Site	F	F_1-a/2_	F_a/2_	Conclusion
Huayuankou station	0.50	0.39	3.85	Acceptance
Gaocun station	2.83	0.39	3.85	Acceptance
Lijin station	3.85	0.39	3.85	Acceptance

**Table 4:** The annual proportion of monthly discharge before and after the operation of the Xiaolangdi reservoir.

Month	Huayuankou Station			Gaocun Station			Lijin Station		
	1963–2001 (1)	2002–2021 (2)	(1)–(2)	1963–2001 (3)	2002–2021 (4)	(3)–(4)	1963–2001 (5)	2002–2021 (6)	(5)–(6)
January	3.82	3.88	−0.06	3.64	3.75	−0.11	4.05	3.66	0.38
February	3.69	4.62	−0.93	3.40	4.06	−0.66	3.32	2.92	0.40
March	7.06	8.00	−0.94	6.54	7.58	−1.04	4.63	3.01	1.63
April	7.08	8.44	−1.36	6.73	8.04	−1.31	4.64	4.14	0.50
May	6.61	8.16	−1.54	6.11	7.67	−1.55	4.46	6.23	−1.77
June	5.33	13.85	−8.52	5.23	13.24	−8.01	3.87	12.13	−8.27
July	10.79	12.85	−2.07	11.74	14.03	−2.29	10.42	18.22	−7.80
August	15.03	9.27	5.76	17.17	9.78	7.39	16.62	13.43	3.19
September	15.78	9.70	6.08	16.07	9.70	6.37	17.96	11.89	6.07
October	12.70	9.59	3.11	13.64	10.14	3.49	15.73	11.95	3.78
November	7.24	6.36	0.88	8.50	6.52	1.98	9.20	7.37	1.83
December	4.88	5.30	−0.41	5.20	5.38	−0.17	5.11	5.04	0.07

## Data Availability

Data and materials are available from the corresponding author upon request.

## References

[1] Ma Z.Z., Wang Z.J., Xia T. (2014). Hydrograph-based hydrologic alteration assessment and its application to the Yellow River. J. Environ. Inform..

[2] Bunn S.E., Arthington A.H. (2002). Basic principles and ecological consequences of altered runoff regimes for aquatic biodiversity. Environ. Manag..

[3] Zhang J., Dong Z.R., Sun D.Y. (2010). Complete river health assessment index system based on eco-regional method according to dominant ecological functions. J. Hydraul. Eng..

[4] Poff N.L. (2018). Beyond the natural runoff regime Broad-ening the hydro-ecological foundation to meet environmental flows challenges in a non-stationary world. Freshw. Biol..

[5] Dong Z.R., Zhang J., Zhao J.Y. (2017). Comments upon progress of environmental flows assessments. J. Hydraul. Eng..

[6] Poff N.L., Richter B.D., Arthington A.H. (2010). The ecological limits of hydrologic alteration (ELOHA): A new framework for developing regional environmental flow standards. Freshw. Biol..

[7] Pang L.M. Number of Dams in the World China Power Grid. 1 March 2021. http://www.chinapower.com.cn/qtsj/20210301/54829.html.

[8] Wang Y., Wang D., Wu J. (2015). Assessing the impact of Danjiangkou reservoir on ecohydrological conditions in Hanjiang river, China. Ecol. Eng..

[9] Poff N.L., Schmidt J.C. (2016). How dams can go with the flow. Science.

[10] Poff N.L., Olden J.D. (2017). Can dams be designed for sustainability?. Science.

[11] Ngor P.B., Legendre P., Oberdorffb T. (2018). Flow alteration by dams shaped fish assemblage dynamics in the complex Mekong-3s river system. Ecol. Indic..

[12] Chen X.X., Ye S., Pan H.L. (2022). Analysis of the impact of reservoir operation on the hydrological situation of rivers—Longyangxia and Xiaolangdi reservoirs as examples. China Rural Water Conserv. Hydropower.

[13] Williams G.P., Gordon W.M. (1984). Downstream Effects of Dams on Alluvial Rivers.

[14] Walling D.E., Fang D. (2003). Recent trends in the suspended sediment loads of the world’s rivers. Glob. Planet. Change.

[15] Vorosmarty C.J., Meybeck M., Fekete B. (1997). The potential impact of neo-cauterization on sediment transport by the global network of rivers. Human Impact on Erosion and Sedimentation(Proc Rabat Symposium. April 1997).

[16] Song X., Zhuang Y., Wang X. (2020). Analysis of Hydrologic Regime Changes Caused by Dams in China. Hydrol. Eng..

[17] LI Y., Chang J., Tu H. (2016). Impact of the Sanmenxia and Xiaolangdi Reservoirs operation on the hydrologic regime of the lower Yellow River. J. Hydrol. Eng..

[18] Kong D.X., Latrubesse E.M., Miao C.Y. (2020). Morphological response of the Lower Yellow River to the operation of Xiaolangdi Reservoir, China. Geomorphology.

[19] Shang H.X., Zheng Y.S., Zhang X.H. (2008). Effects of reservoir utilization on water and sediment conditions in the Ningmeng River. People’s Yellow River.

[20] Miao C.Y., Kong D.X., Wu J.W. (2016). Functional degradation of the water-sediment regulation scheme in the lower Yellow River: Spatial and temporal analyses. Sci. Total Environ..

[21] Wang Y.J., Wu B.S., Zhong D.Y. (2020). Simulating cross-sectional geometry of the main channel in response to changes in water and sediment in Lower Yellow River. J. Geogr. Sci..

[22] Kong D.X., Miao C.Y., Wu J.W. (2015). The hydro-environmental response on the lower Yellow River to the water-sediment regulation scheme. Ecol. Eng..

[23] Hu C.H., Chen J.G., Guo Q.C. (2008). Regulation of water and sediment processes in the Yellow River and shaping of the downstream channel in the water channel. J. Tianjin Univ..

[24] Li M.C., Hu C.H. (2012). Multi-timescale analysis of water-sediment and flatland flow in the lower Yellow River. J. China Inst. Water Res. Hydropower Res..

[25] Zhang Y.Y., Zhong D.Y., Wu B.S. (2012). Multi-timescale phenomena of runoff in the Yellow River Flats. Adv. Water Sci..

[26] Liu X.Y., Ma S.Y., Dang S.Z. (2017). Changes in sediment production situation in the Yellow River basin in the past 100 years. Sedim. Res..

[27] Liu X.Y., Gao Y.F., Ma S.B. (2018). Sediment reduction effect of silt dams on the Loess Plateau and their temporal effects. J. Water Res..

[28] Zhou Y.Y., Huang H.Q., Ran L.S. (2018). Hydrological controls on the evolution of the Yellow River Delta: An evaluation of the relationship since the Xiaolangdi Reservoir became fully operational. Hydrol. Process..

[29] Li J., Chu M.H., Zhang Y., Zhu C.H. (2022). Characteristics of the evolution of continental beaches in the wandering section of the lower Yellow River from 1986–2018. People’s Yellow River..

[30] Esri “Topographic” [Basemap]. Scale Not Given. “World Topographic Map”. 19 February 2012. http://www.arcgis.com/home/item.html?id=30e5fe3149c34df1ba922e6f5bbf808f.

[31] Wang Y.J., Wu B.S., Zhong D.Y. (2020). Adjustment in the main-channel geometry of the lower Yellow River before and after the operation of the Xiaolangdi Reservoir from 1986 to 2015. J. Geogr. Sci..

[32] Tang Q.C., Li X.Y. (1982). Calculation and discussion on the non-uniform coefficient of annual runoff distribution. Res. Sci..

[33] Wang H.X., Zhao Y.Y., Liu J.H. (2020). Water and sediment regime evolution and its influencing factors in the Lower Yellow River in recent 50 years. J. Hydroel. Power.

[34] He W., Bu R.C., Xiong Z.P. (2013). Variation trend of temperature and precipitation in Northeast China during 1961–2005. Acta Ecol. Sin..

[35] Xue J., Xia Z.Q., Huang F. (2013). Influence of large reservoirs on interannual variation and annual distribution of downstream river runoff. Hydropower Energy Sci..

[36] Chen Y.B., Zhang X., Yang W.F. (2021). Influence on river runoff quantitative analysis of construction operation of cascade reservoirs. People’s Yangtze River.

[37] Liu C.M., Wang K.W., Wang M. (2022). Runoff and its influencing factors in the Yellow River Basin from 1956 to 2016. People’s Yellow River.

[38] Chong K.L., Huang Y.F., Koo C.H. (2022). Spatiotemporal variability analysis of standardized precipitation indexed droughts using wavelet transform. J. Hydrol..

[39] Zhang J.P., Xiao H.L., Fang H.Y. (2022). Component-based Reconstruction Prediction of Runoff at Multi-time Scales in the Source Area of the Yellow River Based on the ARMA Model. Water Res. Manag..

[40] Labat D. (2005). Recent advances in wavelet analyses: Part 1. A review of concepts. J. Hydrol..

[41] Zhang H.B., Huang Q., Zhang Q. (2016). changes in the long-term hydrological regimes and the impacts of human activities in the main Wei River, China. Hydrol. Sci..

[42] Anuragi A., Singh S.D. (2022). EEG-based cross-subject emotion recognition using Fourier-Bessel series expansion based empirical wavelet transform and NCA feature selection method. Inf. Sci..

[43] Yao W.Y., Gao Y.J., An C.H. (2015). Trend analysis of 100-year scale water and sediment variation in the upper and middle reaches of the Yellow River. Adv. Water Res. Hydropower Sci. Technol..

[44] Yu L., Wang G.H., Zou Z.K. (2016). Adjustment pattern of riverbed cross-sectional morphology in the Shashi River section of the Upper Jingjiang River. J. River Sea Univ..

[45] Wang W.S., Ding J., Xiang H.L. (2002). Wavelet transform method for multi-time scale analysis of hydrological time series. J. Sichuan Univ..

[46] Li Z.H., Wang H., Han G.D. (2007). Progress in the study of the Yellow River downstream flow break. Ecol. Environ..

[47] Shang W.X., Peng S.M., Wang Y. (2022). Analysis of the impact of Xiaolangdi hydrological hub on the ecology of the lower reaches of the Yellow River. Water Res. Conserv..

[48] Guo Q.C., Hu C.H., Cao W.H. (2005). Desilting effect of large reservoirs in the middle and lower reaches of the Yellow River on the downstream river channel. J. Water Res..

